# Approach for quick exploration of highly effective broad-spectrum biocontrol strains based on PO8 protein inhibition

**DOI:** 10.1038/s41538-023-00210-5

**Published:** 2023-09-01

**Authors:** Mei Gu, Jiayun Fu, Honglin Yan, Xiaofeng Yue, Shancang Zhao, Qi Zhang, Peiwu Li

**Affiliations:** 1 Oil Crops Research Institute, Chinese Academy of Agricultural Sciences, Wuhan, 430061 China; 2 Key Laboratory of Biology and Genetic Improvement of Oil Crops, Wuhan, 430061 China; 3 Hubei Hongshan Laboratory, Wuhan, 430061 China; 4 Institute of Quality Standards and Testing Technology for Agro-products, Shandong Academy of Agricultural Sciences, Jinan, 250100 P. R. China; 5 Institute of Food Safety, Hubei University, Wuhan, 430061 China; 6 Ministry of Agriculture and Rural Affairs and Key Laboratory of Detection for Mycotoxins, Wuhan, 430061 China; 7 Xianghu Laboratory, Hangzhou, 311231 P. R. China

**Keywords:** Food microbiology, Environmental microbiology

## Abstract

Aflatoxin is a group of strongly toxic and carcinogenic mycotoxins produced by *Aspergillus flavus* and other *Aspergillus* species, which caused food contamination and food loss problems widely across the world especially in developing countries, thus threatening human health and sustainable development. So, it is important to develop new, green, and broad-spectrum biocontrol technology for the prevention of aflatoxin contamination sources. Previously, we found that the PO8 protein from aflatoxigenic *A. flavus* could be used as a biomarker to predict aflatoxin production in peanuts (so the PO8 is named as an early warning molecule), which infers that the PO8 is relative to aflatoxin production. Therefore, in the study, based on inhibiting the PO8, a new and quick strategy for screening aflatoxin biocontrol strains for developing control agents was presented. With the PO8 inhibition method, four biocontrol strains (2 strains were isolated from peanut kernels with sterilized surface and another 2 strains from peanut rhizosphere soil) were selected and combined to increase prevention wide-spectrum. As a result, the combination showed over 90% inhibition to all tested aflatoxigenic *A. flavus* isolated from three different peanut production areas (north, middle, and south areas of China), and better than any single strain. The field experiments located in five provinces of China showed that the practice prevention effects (inhibition of aflatoxigenic fungi on the surface of the peanuts) were from 50% to over 80%. The results indicated that the strategy of inhibiting the early warning molecule PO8 can be used to develop aflatoxin control agents well.

## Introduction

Aflatoxins, derivatives of polyketide, secondary metabolites primarily produced by fungal species from the Aspergillus genus, especially, Aspergillus flavus and Aspergillus parasiticus, are a group of structurally related toxic, mutagenic, and carcinogenic mycotoxins^1–3^. Among the many analogs and derivatives of aflatoxins that have been identified, the B-series (aflatoxins B1 and B2), the G-series (aflatoxins G1 and G2), and aflatoxin M1 have been known for their hazard to humans. Especially, aflatoxin B_1_ (AFB_1_), the most toxic and commonly occurring one, has been classified as a group I human carcinogen by the International Agency for Research on Cancer (IARC). The contamination of food and feed caused by aflatoxins is not only high in incidence and prevalence worldwide but also extremely harmful. For instance, aflatoxins contaminated peanuts, corn, cotton, tree nuts, and other susceptible crops both in the field and during the storage including pre- and post-harvest, or processing^3,4^, and they are increasingly detected in China^5^, Italy, and southern Europe^6^. Furthermore, Lee and Ryu (2017) summarized that 45% of global corn and 33% of corn-based products were contaminated by aflatoxins^7^. Moreover, aflatoxins are even associated with occasional outbreaks of acute aflatoxicosis, which lead to death shortly after exposure to aflatoxin^6^.

To control the contamination and harm caused by aflatoxins, the fundamental measure is to control the aflatoxigenic *A. flavus*, because aflatoxins are produced by aflatoxigenic *A. flavus*. Basic preventive measures such as Good Agricultural Practices (GAPs) and Good Manufacturing Practices (GMPs) as aflatoxin preventive measures have proven effective when combined with proper physical strategies (field management, physical separations, and moisture controls) and chemical strategies (fungicides and chemical absorbents)^8^. However, in most cases, the physical and chemical methods were inefficient, due to a nutritional loss of the processed foods, difficulty in removing residues of the toxic compounds, or the development of resistant biotypes of pathogens^9^. In recent years, biological control using native microorganisms or their metabolites to inhibit the growth of aflatoxigenic *A. flavus* and prevent the production of aflatoxin has recently emerged as a promising alternative because it is harmless to mammals and pollution-free to the environment^10^. Previous studies have extensively explored mycotoxin reduction using *Bacillus* species^11–16^. Furthermore, Atehnkeng et al., (2014) conducted research on applying aflatoxigenic *A. flavus* to prevent aflatoxin contamination in maize^17^. Biological control has been regarded as a more environmentally friendly and safer method, which can be carried out generally at pre- and or post-harvest that mainly focuses on the removal of aflatoxin.

It is of great significance to develop a reliable prediction method, which contributes to preemptively preventing mycotoxins contamination. To develop the source control of toxin-producing fungi, our team previously found that the PO8 protein from aflatoxigenic *A. flavus* could predict the risk of toxin contamination before the production of aflatoxins, and was named as an early warning molecule^18,19^. And then, we proposed a new idea: is it feasible to screen and develop control agents by inhibiting early warning molecules? To answer the above question, this paper takes the prevention and control of peanut aflatoxin as an example and successfully reveals that it is feasible to screen and develop prevention and control strategies by inhibiting early warning molecules PO8. Therefore, this paper provides a new way for the development of new inhibitors of aflatoxin.

## Results

### Expression and production of PO8 in *A. flavus* strains

**Fig. 1:**
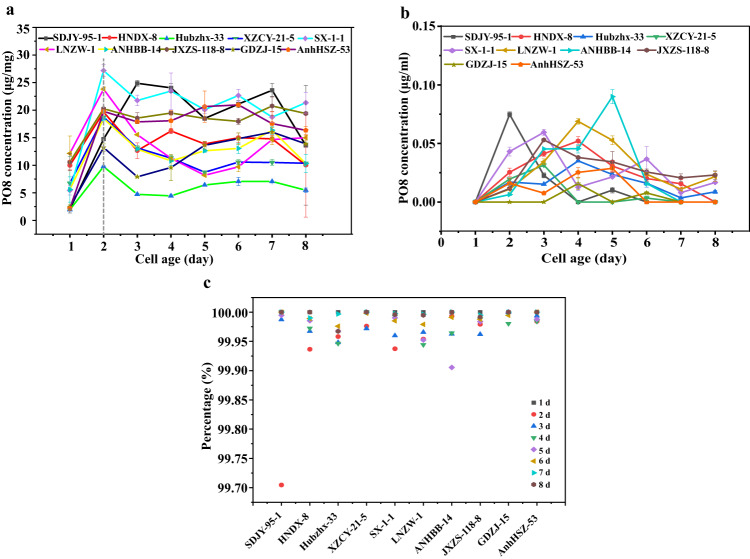
The occurrence regularity of intracellular and extracellular PO8 in ten aflatoxigenic *A. flavus* strains from different peanut-producing areas in China. **a** Intracellular PO8 content at different times. **b** Extracellular PO8 content at different times. **c** The percentage of intracellular PO8 to the total PO8 content. The error bars indicate standard deviations.

Our previous research has made it clear that the early warning molecule PO8 can be detected before the production of aflatoxin, which can predict the contamination risk before the production of the toxin, and has established the ELISA detection method for the protein PO8^20^. However, to quickly and accurately quantitatively detect this molecule in later studies, it is necessary first to ascertain whether this protein mainly exists inside or outside the cell after expression. In the present study, ten aflatoxigenic *A. flavus* strains LNZW-1, SX-1-1, SDJY-95-1, ANHBB-14, AnhHSZ-53, Hubzhx-33, JXZS-118-8, XZCY-21-5, HNDX-8, and GDZJ-15 from ten peanut production in China were selected randomly. The ten strains were cultured in Liquid Sabouraud Medium for 8 days, and samples were collected every day to determine the content of intracellular and extracellular PO8. The result showed that the contents of intracellular PO8 from every strain grew rapidly on the second day and reached their peak production (Fig. 1a). Subsequently, they entered into a stable and stagnant period, and their contents remained at the level of 48 h. The results also showed that the extracellular PO8 reached its peak contents ranging from the cultures for 2 days to 5 days among the different strains (Fig. 1b).

Comparing the PO8 contents of intracellular and extracellular, as shown in Fig. 1c, the percentage of intracellular PO8 was more than 99% for 8 days. Therefore, this result indicated that the incubation time of the strains should be within 48 h and intracellular PO8 content merely needed to be observed if we continued to conduct the PO8-related inhibition test study.

### Screening of biocontrol bacterial strains based on PO8 inhibition

**Fig. 2:**
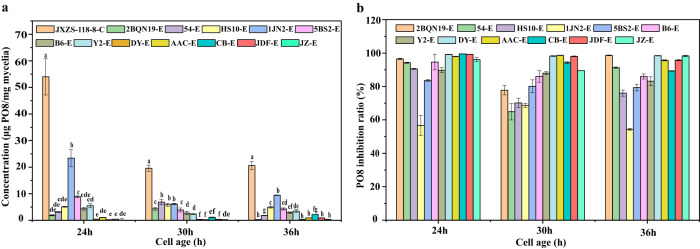
Screening of biocontrol bacterial strains based on PO8 inhibition, “C” represents the control group, and “E” represents the experimental group. **a** Changes of PO8 in the mycelia of a high aflatoxin-producing capacity A. flavus strain JXZS-118-8 under the inhibition conditions of 12 biocontrol bacteria. **b** The inhibition ratio of PO8 in A. flavus strain JXZS-118-8 under the inhibition conditions of 12 biocontrol bacteria. Values with different letters indicate significant differences (*p* < 0.05). The error bars indicate standard deviations.

To screen biocontrol bacteria strains with the best inhibitory effect, a total of 12 strains of biocontrol bacteria were screened by inhibiting the expression of PO8 in *A. flavus* JXZS-118-8 with high aflatoxin-producing capacity in the present study. The result (Fig. 2a) showed that compared to the control group, the concentrations of PO8 in all bacterial treatment groups were significantly (*p* < 0.05) reduced at three different incubation times. In particular, of the 12 treatment groups, five of twelve bacteria (DY-E, AAC-E, CB-E, JDF-E, and JZ-E) effectively inhibited the secretion of PO8, and the differences were significant in the case of treatments of DY-E, AAC-E, CB-E, and JDF-E.

Correspondingly, as shown in Fig. 2b, at 24 h, 30 h, and 36 h, the highest inhibition ratios were the DY-E group (99%, 95%, 98%), followed by the JDF-E group (99%, 98%, 95%), AAC-E group (97%, 98%, 95%), CB-E group (99%,94%, 85%), JZ-E group (96%, 89%, 98%). In the other experimental groups, the inhibitory effects were relatively poor, for example, 2BQN19-E (96%, 77%, 98%), 54-E (94%, 64%, 91%), HS10-E (90%, 70%, 76%), 1JN2-E (56%, 68%, 54%), 5BS2-E (83%, 80%, 79%), B6-E (94%, 86%, 85%), Y2-E (89%, 87%, 83%). In other words, the inhibition rates of five kinds of bacteria were more than 90%, including DY, AAC, CB, JDF, and JZ, these five were significantly more effective than the other bacterial strains.

### Broad-spectrum determination of the biocontrol strains against PO8 and aflatoxin in *A. flavus* strains

**Fig. 3:**
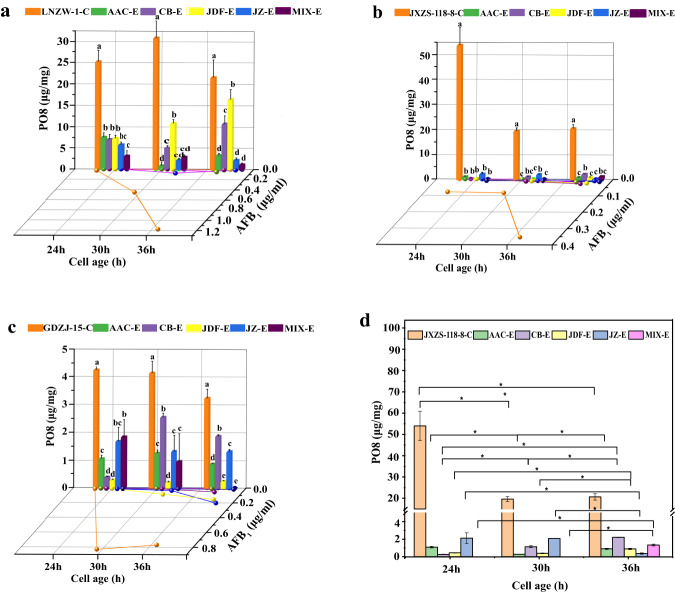
Effect of four biocontrol bacteria and their mixture on PO8 and AFB_1_ in three *A. flavus* strains, and “C” represents the control group, “E” represents the experimental group. **a** LNZW-1, **b** JXZS-118-8, **c** GDZJ-15, **d** the statistical significance of the effect of four biocontrol bacteria and their mixture on PO8 in *A. flavus* strain JXZS-118-8 among different culture times. Values with different letters indicate significant differences (*p* < 0.05), and * represents a significance level of *p* < 0.05. The error bars indicate standard deviations.

To determine the broad spectrum of the prevention and control effect of the four biocontrol strains and all their mixture, we chose three aflatoxigenic *A. flavus* strains LNZW-1 from east-north of China, JXZS-118-8 from the middle of China, and GDZJ-15 from south of China for the test. Briefly, we made the four biocontrol bacteria AAC, CB, JDF, JZ, and four of their mixture to inhibit *A. flavus* strains LNZW-1, JXZS-118-8, and GDZJ-15 in the present study, respectively. And then, we monitored the amounts of PO8 and AFB_1_ at different incubation times, to clarify the PO8 and AFB_1_ in *A. flavus* under bacteria-induced stress. The result of prevention *A. flavus* strain LNZW-1 is shown in Fig. 3a, in which PO8 was almost completely inhibited by the mixture at 36 h, although the inhibition rates by CB-E and JDF-E were not good. As to the prevention of aflatoxin, all five treatments almost completely inhibited the production of aflatoxin. The biocontrol effects of the bacteria on *A. flavus* strain JXZS-118-8 are shown in Fig. 3b, the result showed that aflatoxin and PO8 were almost completely inhibited in all treatments. The result of prevention of *A. flavus* strain GDZJ-15 is shown in Fig. 3c and revealed that both PO8 and aflatoxin were almost completely inhibited by the mixture at 36 h, although the inhibitions of PO8 by CB-E and JZ-E were not good and the inhibition of aflatoxin by JZ-E was not good. Further, in Fig. 3d, the results of the statistical significance of the effect of four biocontrol bacteria and their mixture on PO8 in JXZS-118-8 *A. flavus* strain between different culture times are shown. As we can see, at different culture times, the difference in the inhibitive effect of the four species and their mixtures at different incubation times was significant (*p* < 0.05). However, the differences in the inhibitive effects of AAC-E between 24 h and 36 h, JDF-E, JZ-E, and MIX-E between 24 h and 30 h were not significant.

The above results indicated that by mixing the four biocontrol bacteria, the biocontrol spectrum was effectively widened. And it infers that the mixture could be used to prevent and control aflatoxin contamination in different areas.

### Peanut inoculation experiment

**Fig. 4:**
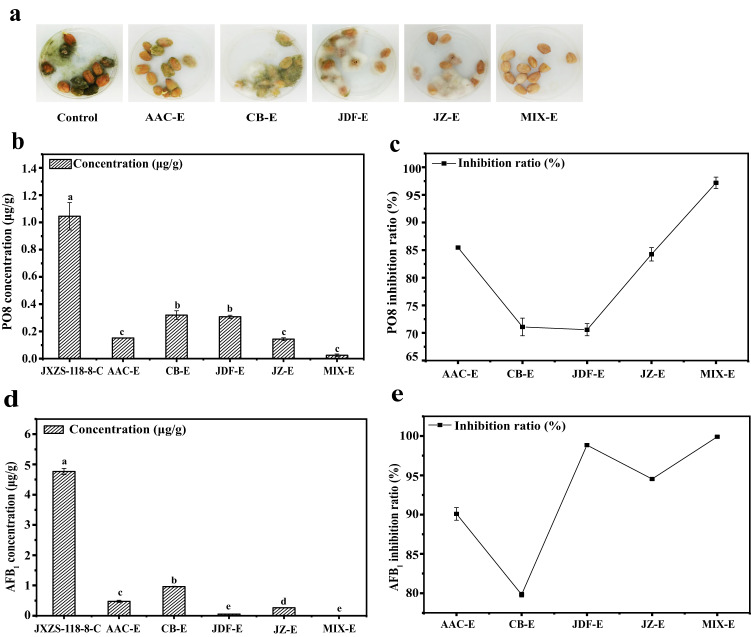
The effects of four biocontrol bacteria and their mixture on peanuts inoculated by *A. flavus*, “C” represents the control group, and “E” represents the experimental group. **a** *A. flavus* grew in peanut kernels (control group) and peanut kernels + the bacterial suspension of AAC, CB, JDF, JZ and their mixture (experiment group); effects of four biocontrol bacteria and their mixture on the concentration of PO8 (**b**) and the inhibitive ratio of PO8 (**c**) in the different treatment groups; effects of four biocontrol bacteria and their mixture on the concentration of AFB_1_ (**d**) and the inhibition rate of AFB_1_ (**e**) in the different treatment groups. Different letters indicate a significant difference (*p* ≤ 0.05). The error bars indicate standard deviations.

Biocontrol efficacy was evaluated in the peanut inoculation experiment. As shown in Fig. 4a, we observed that *A. flavus* spores were significantly reduced in five bacterial treatments, and the control group produced more spores compared with the experimental groups after 7 days of incubation at 28 °C in darkness. The mixed experimental group was the most effective treatment, resulting in a minimal amount of spores, followed by JDF-E, JZ-E, AAC-E, and CB-E. In addition, JDF-E, and JZ-E did not present extensive visual contamination with *A. flavus* spores in comparison with other experimental groups. Especially, clear macroscopic differences could be observed on the peanut kernels treated with AAC, CB, and the mixture group. In addition, we monitored the changes in the concentration of PO8 and AFB_1_ in peanut kernels inoculated with *A. flavus* under the separated action of the four screened biocontrol bacteria and the combined action of their mixed bacterial solutions.

As shown in Fig. 4b, c, the concentration of PO8 in five treatment groups was significantly different (*p* < 0.05), among the five treatment groups, the PO8 content was the lowest and the inhibition rate was the highest in the mixed inhibition group.

A similar pattern was found in the AFB_1_ analysis. As shown in Fig. 4d, e, detectable levels of AFB_1_ were significantly (*p* < 0.05) reduced by treatment with the four biocontrol bacteria and their mixture of the bacterial suspension. The mixed experimental group showed to be the most effective treatment, reducing final AFB_1_ concentration with the highest inhibition rate (99.9%).

### Field trial

**Fig. 5:**
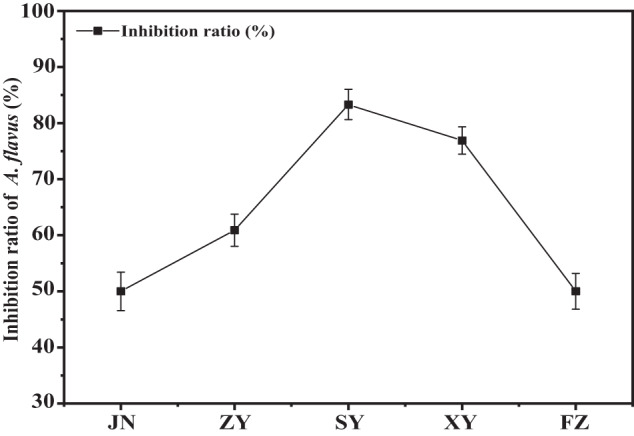
The inhibitive effects of biocontrol agents BBBE on *A. flavus* in peanut rhizosphere soils of five demonstration sites JN, ZY, SY, XY, and FZ. The error bars indicate standard deviations.

The prevention and control effect of the biocontrol agents BBBE which was made from the four biocontrol bacteria strains screened out was also put into practical application in test fields. As shown in Fig. 5, the Plate counting method was used to determine the abundance of *A. flavus* in peanut rhizosphere soil. The results showed that BBBE exhibited different inhibition effects on *A. flavus* in five regions, and the inhibition rate ranged from 50% to 83.3%. Among them, BBBE exhibited the highest inhibition rate (83.3%) in SY, followed by XY (76.9%), ZY (60.9%), JN (50%), and FZ (50%). The result showed that BBBE had a good inhibitory effect on *A. flavus* in peanut rhizosphere soil.

## Discussion

To prevent and control the contamination caused by aflatoxins from their source, the growth of aflatoxigenic A. flavus must be controlled fundamentally. Previously, in our laboratory, we discovered the early warning molecule PO8, the metabolites with higher concentration, whose presence suggested the presence of aflatoxigenic *Aspergillus* species in agricultural products.

Here, we initially explored the use of the early warning molecule PO8 to develop biological agents for aflatoxin contamination in peanuts. Hence, we first monitored the secretion rule of PO8, which laid the foundation for subsequent research. We selected 12 biocontrol bacteria from the biocontrol strain library in our laboratory to monitor the inhibitory effect on PO8 and screened out 5 strains (DY- *Bacillus licheniformis*, AAC-*Enterobacter ludwigii*, CB-*Brevibacillus laterosporus*, JDF-*Bacillus amyloliquefaciens*, JZ-*Bacillus mucilaginosus*) with relatively good inhibitory effects. Of the five species, AAC and JDF showed consistently better inhibition effects on PO8, and DY, CB, as well as JZ showed similar inhibition effects. In the follow-up study, why did we abandon DY and choose the remaining four species of bacteria? Because previous studies reported that chitinase produced by *Bacillus licheniformis* had the potential for cell wall lysis of many phytopathogenic fungi^21^, meanwhile, had reversible effects on fungal morphology^22^. What’s more, there are few reports on the use of B. licheniformis as a biofertilizer to prevent and control the growth of A. flavus in the field (in the Web of Science). *Brevibacillus laterosporus* has long been noted for its broad-spectrum antifungal properties when isolated from rhizosphere soil samples^22^, which has been associated with the production of a wide range of enzymes and antibiotics. Ghazanchyan et al., (2018) reported that *Brevibacillus laterosporus* is reported to strongly inhibit the growth of numerous apple phytopathogens (such as *Rhizoctonia solani*, *Fusarium oxysporum*, *Fusarium solani*, and *Physalospora piricola*) and suppress potato common scab by reducing pathogen abundance and regulating soil bacterial community^23,24^. *Bacillus mucilaginosus*, known as potassium bacteria or silicate bacteria, is an important functional bacteria widely used in microbial fertilizers^25^. It can promote the conversion of soil ineffective phosphorus and potassium, increase the supply of soil phosphorus and potassium, and then promote plant growth, and improve crop yield and quality^26^. A recent study reported that *B. mucilaginosus* played a very important role in plant growth, and total N and chlorophyll contents are improved under the application of phosphate-solubilizing bacteria *B. subtilis* and *B. mucilaginosus*^27^. Taking all these into consideration, we chose AAC, CB, JDF, and JZ four bacteria for further research.

We continued to explore the potential biocontrol capabilities of the four bacterial isolates towards three strains of aflatoxigenic *A. flavus* strains with high aflatoxins-producing capacity (APC) from Northern, Central, and Southern China. In this study, all four isolates presented promising antagonistic characteristics in inhibiting the production of PO8 and AFB_1_. And it indicated that the competition between the biocontrol bacteria and *A. flavu*s directly impaired *A. flavus’s* vegetative development, which is responsible for nutrient uptake and physical proliferation^28^.

In this study, four biocontrol bacteria strains including AAC-*Enterobacter ludwigii*, CB-*Brevibacillus laterosporus*, JDF-*Bacillus amyloliquefaciens*, JZ-*Bacillus mucilaginosus*, among them, *Enterobacter ludwigii* had a clear and stable inhibitory effect on PO8 (more than 69–95%) and AFB_1_ (more than 95–100%). At present, there is no report on the inhibition of *A. flavus* by *Enterobacter ludwigii* (in Web of Science). Previously, we have found that *Enterobacter ludwigii* has a strong antibacterial ability to inhibit *A. flavus* with a high inhibitory effect (90.5%) and applied for a patent^29^. The results of this research are consistent with our previous research results. The remaining three bacteria belong to the *Bacillus* species, which is one of the main producers of antifungal compounds, are capable of resisting harsh environmental changes and still being able to grow and inhibit fungal growth due to their spore-forming ability^30–32^. Additionally, Bluma and Etcheverry (2006) found that a reduction of AFB_1_ levels in maize grains treated with candidate strains of *B. amyloliquefaciens*^33^. Etcheverry et al. (2009) also reported a reduction of *A. flavus* growth after utiliazation of *Bacillus amyloliquefaciens* on maize ears^34^. Until now, there is no report on the biocontrol effect of *Brevibacterium lateralis* and *Bacillus mucilaginosus* on *A. flavus*. In the present study, *Brevibacillus laterosporus*, *Bacillus amyloliquefaciens*, and *Bacillus mucilaginosus* had different inhibitory effects on PO8 and AFB_1_, and the control effect is poor when individually co-cultured with *A. flavus*. However, the mixture of four strains showed a good inhibitory effect on PO8 and AFB_1_ in three strains of *A. flavus* with more than 94% (94–100% at 36 h). Our results indicated that the biocontrol bacteria screened out had a defect in the broad spectrum of control, but the mixture exhibited broad-spectrum prevention and control potential.

To further verify the inhibitory effect of the four biocontrol bacteria on *A. flavus*, we carried out the peanut inoculation experiment. On the whole, when *A. flavus* strains were inhibited by the four biocontrol bacteria individually, the treatment groups showed different inhibition effects on *A. flavus*. And the inhibitory effect of the mixed treatment was much better, as well as no hyphae and spores of *A. flavus* were observed in the plate. The difference between control group and treatment groups was significant (*p* < 0.05) in PO8 and AFB_1_, and the inhibition rate reached more than 95%. In general, the inhibition effect of the mixed experimental group is the best.

In the field experiment, according to the results of the inhibitive effects of BBBE on the abundance of *A. flavus* in peanut rhizosphere soils of five demonstration sites such as JN, ZY, SY, XY, and FZ, the control effect of the mixed microbial agent on *A. flavus* on peanut surface was from 50% to 83.3%. Our results showed that the biocontrol agent developed in this study applied for aflatoxin prevention in peanut fields with high efficiency and broad-spectrum, and the method to screen the biocontrol bacteria based on PO8 inhibition was scientific and feasible. Therefore, our study established a new approach for developing aflatoxin biocontrol strains based on PO8 protein inhibition and also provided new technology for aflatoxin source control, food safety, and sustainable development.

## Methods

### Strains and culture conditions

**Fig. 6:**
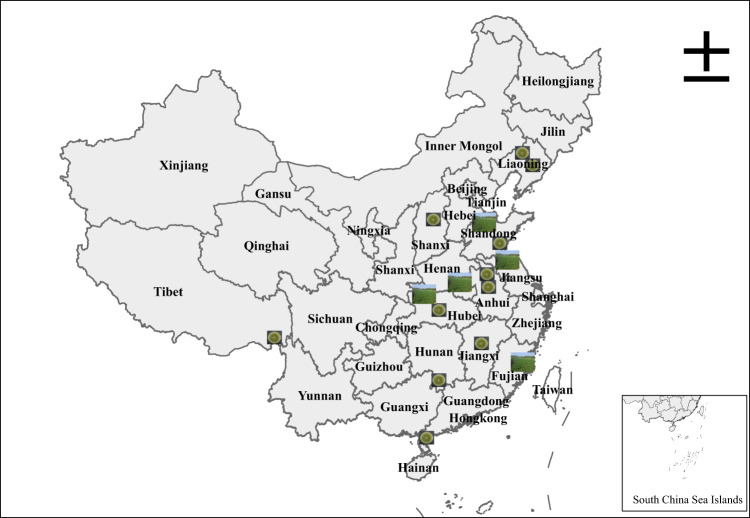
The source locations of *A. flavus* strains used and the distribution map of the field test in this research. In the map, the picture “plate” represents the source locations of *A. flavus* strains used in this work, and the picture “peanut field” represents the demonstration sites of the biocontrol agent BBBE used in field trials. In this figure, 1 cm represents a distance of 230 km in the field.

The aflatoxigenic *A. flavus* strains LNZW-1, SX-1-1, SDJY-95-1, ANHBB-14, AnhHSZ-53, Hubzhx-33, JXZS-118-8, XZCY-21-5, HNDX-8, and GDZJ-15 were isolated from the peanut soils in Liaoning, Shanxi, Shandong, Anhui, Hubei, Jiangxi, Xizang, Hunan and Guangdong peanut-planting provinces of China, which is depicted in Fig. 6. The *A. flavus* strains were grown on DG-18 agar medium (protein 5 g, glucose 10 g, potassium dihydrogen phosphate 1.0 g, magnesium sulfate 0.5 g, zinc sulfate 0.01 g, copper sulfate 0.005 g, ammonium chlortronitol 0.002 g, gildamycin hydrochloride 0.05 g, agar 15 g, and 1 L water, pH adjusted to 5.6 ± 0. 2, 121 °C sterilized for 20 min). The conidia were harvested with a 0.01% Tween-80 solution after 5–6 days of culture at 28 °C in darkness. The biocontrol bacteria including 2BQN19 (*Stenotrophomonas* sp.), 54 (*Bacillus amyloliquefaciens*), HS10 (*Bacillus licheniformis*), 1JN2 (*Bacillus subtilis*), 5BS2 (*Bacillus subtilis*), B6 (*Bacillus* sp.), Y2 (*Bacillus cereus*), DY (*Bacillus licheniformis*), AAC (*Enterobacter ludwigii*), CB (*Brevibacillus laterosporus*), JDF (*Bacillus amyloliquefaciens*), JZ (*Bacillus mucilaginosus*), selected from the biocontrol strain library in our laboratory, were isolated from peanut or their rhizosphere soil, which were previously reported by Yang^35^, Li et al. (2019)^29^, and Zhang et al. (2021)^36^. The biocontrol bacteria strains were cultured at 28 °C for 18–24 h in an LB liquid medium, which contained 5 g of yeast extract, 10 g of peptone, and 10 g of NaCl per liter. *A. flavus* and the bacterial antagonists, respectively, were prepared by quantifying the conidia and bacterial cells using a hemocytometer.

### The observation of the secretion regularity of PO8 at different times in high virulence-producing *A. flavus* strains

Briefly, ten *A. flavus* strains were grown in conical flasks containing 50 ml Sabouraud’s Dextrose Broth (peptone 10 g, glucose 40 g, 121 °C sterilized for 20 min) with 5 × 10^5^ conidia/mL and shaken (180 rpm) at 28 ± 1 °C for 8 days. The mycelium was collected by filtration at different incubation times every 24 h under sterile conditions with sterile water and gauze, and then quick-frozen in liquid nitrogen, and freeze-dried for further use. The culture medium was also collected into the sterile tubes and then stored at −20 °C for use. Three biological replicates were set up for all control and experimental groups.

### Screening of biocontrol bacterial strain based on PO8 inhibition

Briefly, the *A. flavus* strain JXZS-118-8 was grown in conical flasks containing 50 ml Sabouraud’s Dextrose Broth with 5 × 10^5^ conidia/mL and shaken (180 rpm) at 28 °C for 12 h. And then, 12 biocontrol bacteria strains were added to the above conical flasks to reach a final concentration of 10^7^CFU/mL and continued to culture for 36 h. At different incubation times (24 h, 30 h, and 36 h), the mycelia were collected into the sterile tubes and then stored at −20 °C. Three biological replicates were set up for all experimental and control groups.

Briefly, the *A. flavus* strains LNZW-1, JXZS-118-8, and GDZJ-15 were grown in conical flasks containing 50 ml Sabouraud’s Dextrose Broth with 5 × 10^5^ conidia/mL and shaken (180 rpm) at 28 ± 1 °C for 12 h. And then, the suspension of four biocontrol bacteria strains (AAC, CB, JDF, JZ,) and their mixture (AAC + CB + JDF + JZ) was added to the above conical flasks to reach a final concentration of 10^7^ CFU/mL and continued to culture for 36 h. At different incubation times (24 h, 30 h, and 36 h), the mycelia that were collected for the detection of PO8 by being filtered out of the liquid medium with sterile gauze and washed three times with sterilization water to separate mycelia from bacterial cells, and Sabouraud’s Dextrose Broth were collected into the sterile tubes and then stored at −20 °C for the detection of AFB_1_. Three biological replicates were set up for all experimental and control groups.

### Peanut Inoculation experiment

Healthy postharvest mature peanut seeds were selected for the experiments to investigate the phenotypic data of the peanut matrices. All seeds were surface-sterilized by immersion in 70% ethanol for 1 min and rinsed with sterile distilled water three times for 1 min each, and then the moisture on the peanut surface was absorbed by sterilization filter paper. Two hundred microliters of the conidial suspension (5.0 × 10^5^ CFU/mL) were added to the experimental and control plates, respectively. In the experimental group, 3 mL of bacterial suspension of the AAC, CB, JDF, JZ, and their mixture were added to the 10.0 g peanut seeds in a sterile Petri plate (ensure that the peanut surface is completely covered). In the control group, 3 mL of ultrapure water was added to the peanut seeds in sterile Petri plates. Then, the experimental and control group samples were placed in an incubator and cultured at 28 °C in darkness for 7 days. Three biological replicates were set up for all experimental and control groups.

### Field trials

The four biocontrol bacteria strains screened out were mixed to produce biocontrol agents BBBE for use in field trials in 2021. Field trials were conducted at five sites, including Junan County (JN, Shandong Province), Zhengyang County (ZY, Henan Province), Siyang County (SY, Jiangsu Province), Xiangyang County (XY, Hubei Province), Fuzhou City (FZ, Fujian Province) in 2021. In experimental plots, biological bacteria fertilizer of 30 kg hm^−2^ was added with fertilizer material, the peanut variety was the main local variety, and the local conventional sowing method, as well as management measures, were adopted for weeding pest, and boom control. At the same time, the control plots were set.

### Determination of *A. flavus* abundance in peanut rhizosphere soil

The method to isolate and identify A. flavus was conducted according to the method proposed by Zhang et al.^37^. In detail, using the five-point sampling method, 5 peanut plants were randomly selected within the range of 2 m^2^, and the peanut rhizosphere soil samples were collected, and they were mixed into one sample. A total of 10 samples including five control samples and five treatment samples were collected from each demonstration base. To prepare soil suspensions, 10.0 g soil samples were added to conical flasks containing 90 mL of sterile water on the ultra-clean workbench, and mixed them thoroughly by shaking at 28 °C (200 rpm/min, 2 h). After that, 50 μL of soil suspension was transferred to the sterile plates with DG-18 solid medium and spread evenly using a glass spreader. And these plates were placed in an incubator at 28 °C in darkness. The colonies were observed and counted regularly, and the hyphae were identified by morphological and molecular biology methods. The plates that were contaminated were cleaned up in time to avoid cross-contamination.

The number of aflatoxin-producing A. flavus colonies per gram of soil was calculated by the following formula:

$${\rm{The\; colony\; count}}\left({\rm{CFU}}/{\rm{g}}\right)=\left[{\rm{N}}/\left({{\rm{V}}}_{1}\times {\rm{m}}\right)\right]\times {\rm{V}}$$

Where N is the colony count of the plates, V1 is the volume of injection, m is the mass of the sample, and V is the volume of the soil suspension.

### Detection of PO8 by Sandwich-ELISA

The method of PO8 extraction differs in different samples. The extraction of PO8 in mycelia was as follows: 10 mg mycelium was added into 2 ml tubes, 500 μL of 1 × PBS (0.01 mol/L) buffer, and four steel balls (3 mm in diameter) were added, grounded at 60 Hz for 30 s in beveller, 12,000 rpm, centrifuged for 10 min, and then the supernatant was filtered with a membrane of 0.22 μm (pore size), and stored at −20 °C for further use.

In the peanut inoculation experiment, the PO8 was extracted according to the following protocol: 2.5 g peanut was added into 50 ml tubes, 10 mL of 1 × PBS buffer, and four steel balls (65 mm in diameter) were added, grounded at 2500 rpm for 10 min, 4500 rpm, centrifuged for 10 min, and then the supernatant was filtered with a membrane of 0.22 μm (pore size), and stored at −20 °C for further use.

PO8 quantification was conducted by the method proposed by Wang et al. (2017) with modifications^20^. Sandwich ELISA was developed to quantify PO8 according to the following protocol: 96-well microtiter plates (Corning, NY, USA) were coated with 200 μL/well of capture antibody (PO8-VHH Nano-antibody) in 1 × PBS buffer at a concentration of 3 μg/mL and incubated at 4 °C overnight. Plates were rinsed six times with 350 μL/well of 1 × PBST (1 × PBS containing 0.05% Tween 20), subsequently blocked with 300 μL/well of 3% skimmed milk in 1 × PBST buffer at 37 °C for 2 h. After nine items of washings with 1 × PBST, 200 μL of serially diluted mycelia were added at 37 °C for 1 h; washing cycles were repeated and 200 μL of rabbit polyclonal antibody at a concentration of 2 μg/mL in 1 × PBS was added and incubated at 37 °C for 1 h; after washing, 200 μL HRP-labeled goat anti-rabbit IgG antibody (Solarbio, Beijing, China, 1:5000 dilution) was incubated at 37 °C for 1 h; after nine times washing, 100 μL of TMB solution were incubated at 37 °C for 10 min; the reaction was then terminated by adding 50 μL of 2 M H_2_SO_4_ and the absorbance values were detected at 450 nm using the CMax Plus microplate reader (Molecular Devices, CA, USA). The relative reduction of PO8 was calculated by the following formula:

$${\rm{Inhibition}}\; {\rm{ratio}}\,( \% )=\left({{\rm{C}}}_{{\rm{PO}}8}-{{\rm{E}}}_{{\rm{PO}}8}\right)/{{\rm{C}}}_{{\rm{PO}}8}\times 100 \%$$

Where C_PO8_ is the concentration of PO8 in control groups, E_PO8_ is the concentration of PO8 in experimental groups.

### Detection of AFB_1_ by High-Performance Liquid Chromatography

AFB_1_ extraction was conducted as follows: Briefly, aflatoxins B_1_ in mycelium or peanuts were extracted using 80% Methanol: water = 80:20, the supernatant was purified using a vacuum filtration system with a 25 mm membrane filter (0.22 μm pore size). Quantitative analysis of aflatoxins was performed by Agilent 1100 HPLC, equipped with a fluorescence detector (FLD), and Romer Derivatisation Unit was used in the system.

The HPLC conditions were as follows: chromatographic column: Waters Symmetry C18 5 μm, 4.6 mm × 250 mm, (C18-A analytical column (15 cm × 4.6 mm × 5 μm); injection volume: 10 μl; column temperature: 30 °C; flow rate: 1.0 ml/min; mobile phase: methanol: water = 45:55 (volume ratio); and fluorescence detection wavelength: excitation wavelength 360 nm and emission wavelength 440 nm.

### Statistical analysis

One-way ANOVA was carried out to evaluate any significant difference in the control effect of biocontrol bacteria, using SPSS 26.0. The different letter means different significance levels (*p* < 0.05).

### Supplementary information

nr-reporting-summary

## Data Availability

The raw data reported in this article were deposited in a public repository. 10.6084/m9.figshare.23354255.
