# Supplementary material for: Approach for quick exploration of highly effective broad-spectrum biocontrol strains based on PO8 protein inhibition
Source: NPJ Sci Food. 2023 Sep 1;7:45. doi: 10.1038/s41538-023-00210-5 (PMC10474023; doi:10.1038/s41538-023-00210-5)
Supplement: Supplementary file 1 — nr-reporting-summary [file 41538_2023_210_MOESM1_ESM.pdf]

nature portfolio

Corresponding author(s): Shancang Zhao, Qi Zhang , Peiwu Li
NPJSCIFOOD-00946R2

Last updated by author(s): Jun 14, 2023

Reporting Summary

Nature Portfolio wishes to improve the reproducibility of the work that we publish. This form provides structure for consistency and transparency in reporting. For further information on Nature Portfolio policies, see our Editorial Policies and the Editorial Policy Checklist.

Statistics

For all statistical analyses, confirm that the following items are present in the figure legend, table legend, main text, or Methods section.

n/a	Confirmed	
☐	☒	The exact sample size (*n*) for each experimental group/condition, given as a discrete number and unit of measurement
☐	☒	A statement on whether measurements were taken from distinct samples or whether the same sample was measured repeatedly
☐	☒	The statistical test(s) used AND whether they are one- or two-sided<br>*Only common tests should be described solely by name; describe more complex techniques in the Methods section.*
☐	☒	A description of all covariates tested
☐	☒	A description of any assumptions or corrections, such as tests of normality and adjustment for multiple comparisons
☐	☒	A full description of the statistical parameters including central tendency (e.g. means) or other basic estimates (e.g. regression coefficient) AND variation (e.g. standard deviation) or associated estimates of uncertainty (e.g. confidence intervals)
☐	☒	For null hypothesis testing, the test statistic (e.g. *F*, *t*, *r*) with confidence intervals, effect sizes, degrees of freedom and *P* value noted<br>*Give P values as exact values whenever suitable.*
☒	☐	For Bayesian analysis, information on the choice of priors and Markov chain Monte Carlo settings
☒	☐	For hierarchical and complex designs, identification of the appropriate level for tests and full reporting of outcomes
☒	☐	Estimates of effect sizes (e.g. Cohen's *d*, Pearson's *r*), indicating how they were calculated

Our web collection on statistics for biologists contains articles on many of the points above.

Software and code

Policy information about availability of computer code

Data collection	*Provide a description of all commercial, open source and custom code used to collect the data in this study, specifying the version used OR state that no software was used.*
Data analysis	*Provide a description of all commercial, open source and custom code used to analyse the data in this study, specifying the version used OR state that no software was used.*

For manuscripts utilizing custom algorithms or software that are central to the research but not yet described in published literature, software must be made available to editors and reviewers. We strongly encourage code deposition in a community repository (e.g. GitHub). See the Nature Portfolio guidelines for submitting code & software for further information.

Data

Policy information about availability of data

All manuscripts must include a data availability statement. This statement should provide the following information, where applicable:

- Accession codes, unique identifiers, or web links for publicly available datasets
- A description of any restrictions on data availability
- For clinical datasets or third party data, please ensure that the statement adheres to our policy

The raw data reported in this article were deposited in a public repository. DOI:10.6084/m9.figshare.23354255.

Human research participants

Policy information about studies involving human research participants and Sex and Gender in Research.

Reporting on sex and gender	*Use the terms sex (biological attribute) and gender (shaped by social and cultural circumstances) carefully in order to avoid confusing both terms. Indicate if findings apply to only one sex or gender; describe whether sex and gender were considered in study design whether sex and/or gender was determined based on self-reporting or assigned and methods used. Provide in the source data disaggregated sex and gender data where this information has been collected, and consent has been obtained for sharing of individual-level data; provide overall numbers in this Reporting Summary. Please state if this information has not been collected. Report sex- and gender-based analyses where performed, justify reasons for lack of sex- and gender-based analysis.*
Population characteristics	*Describe the covariate-relevant population characteristics of the human research participants (e.g. age, genotypic information, past and current diagnosis and treatment categories). If you filled out the behavioural & social sciences study design questions and have nothing to add here, write "See above."*
Recruitment	*Describe how participants were recruited. Outline any potential self-selection bias or other biases that may be present and how these are likely to impact results.*
Ethics oversight	*Identify the organization(s) that approved the study protocol.*

Note that full information on the approval of the study protocol must also be provided in the manuscript.

Field-specific reporting

Please select the one below that is the best fit for your research. If you are not sure, read the appropriate sections before making your selection.

☐ Life sciences ☐ Behavioural & social sciences ☒ Ecological, evolutionary & environmental sciences

For a reference copy of the document with all sections, see nature.com/documents/nr-reporting-summary-flat.pdf

Ecological, evolutionary & environmental sciences study design

All studies must disclose on these points even when the disclosure is negative.

Study description	In this study, we developed a new and quick approach to explore such biocontrol strains based on PO8 protein inhibition. The aflatoxin control agents developed by this research can reduce the abundance of aflatoxin-producing fungi in agricultural soils and reduce the risk of aflatoxin occurrence in crop products. It is significant in improving the ecological environment of agricultural soils and enhancing the safety of crop product quality when widely applied.
Research sample	In this study, the strains we used are Aspergillus flavus strains and biocontrol bacteria, including Stenotrophomonas sp., Bacillus amyloliquefaciens, Bacillus licheniformis, Bacillus subtilis, Bacillus cereus, Enterobacter ludwigii, Brevibacillus laterosporus, Bacillus mucilaginosus. In peanut Inoculation and field experiment, we used healthy postharvest mature peanut seeds and the peanut rhizosphere soil as our research samples.
Sampling strategy	At first, when we carry out the work of screening the biocontrol bacteria and detecting the broad-spectrum of the biocontrol bacteria, we flitered the mycelium with sterile gauze and washed with sterilization water three times, in order to get rid of bacteria. In field trials, when we collected soils, we chose peanut rhizosphere soils, because the peanut rhizosphere soils are closely collected to the peanut root, which can directly reflect the inhibition of aspergillus flavus by biological agents. In order to get the typical samples, we used the five-point sampling method, 5 peanut plants were randomly selected within the range of 2 m2, and the peanut rhizosphere soil samples were collected, and they were mixed into one sample.
Data collection	In the study, Mei Gu detected the PO8 with Sandwich ELISA using CMax Plus microplate reader and AFB1 of the mycelium using High Performance Liquid Chromatography, and then processed and analysed the data on computer.
Timing and spatial scale	From August to October of 2020, we got the data of the regularity of PO8, and screened out four biocontrol bacteria with the best inhibitive effect on PO8 of Aspergillus flavus. In December of 2020, we got the data of Broad-spectrum determination of the biocontrol strains against PO8 and aflatoxin in A. flavus strains, and demonstrated in peanut innoculation experiment. In 2021, the biocontrol agent BBBE which was screened out in this research was used in field experiments in five sites of China in 2021. In December of 2021, we got the data of field trials.
Data exclusions	In this study, when we analysed the data, we excluded the data of the PO8 in the culture medium and the data of the AFB1 of the mycelium. Because the content of the PO8 of the culture medium is very low and the contamination on peanut matrics caused by aflatoxins is extracellular, and secreted toxins, so we excluded the data of the AFB1 of the mycelium.
Reproducibility	The aflatoxin control agents developed by this research can reduce the abundance of aflatoxin-producing fungi in agricultural soils and reduce the risk of aflatoxin occurrence in crop products. It is significant in improving the ecological environment of agricultural soils and enhancing the safety of crop product quality when widely applied. And we state that all attempts to repeat the experiment

were successful.

Randomization

We selected the biocontrol strains randomly from biocontrol strain library in our laboratory. when we detected the broad spectrum of the biocontrol bacteria, the aspergillus flavus strains we selected isolated from east-north , middle, and south of China, which represent the three representative geographical regions of China.

Blinding

The inhibition effect of the four biocontrol bacteria which was screened out in this study was good according to the result of the field experiment.

Did the study involve field work? ☒ Yes ☐ No

Field work, collection and transport

Field conditions

Field trials were conducted at five sites, the relevant parameters are as below: Junan County (JN, Shandong Province), the average annual temperature is 14.6 degrees and the annual precipitation is 1,353.1 mm; Zhengyang County (ZY, Henan Province),the annual temperature is 14.9 degrees the annual rainfall is 936.2mm; Siyang County (SY, Jiangsu Province), the annual temperature is 14.2 degrees and the annual rainfall is 906mm; Xiangyang County (XY, Hubei Province), the annual temperature is 13~21 degrees and the annual rainfall is 420mm; Fuzhou City (FZ, Fujian Province), the annual temperature is 20.6 degrees and the annual rainfall is 1224.2mm

Location

In this study, field trials were conducted at five sites in 2020, including Junan County (JN, Shandong Province,longitude: E 118.83 latitude: N 35.18",the average altitude is 200 metres); Zhengyang County (ZY, Henan Province,longitude: E 114.39, latitude: N 32.61; the average altitude is is 78.5 metres); Siyang County (SY, Jiangsu Province, longitude: E 118.68, latitude: N 33.73 ; the average altitude is 13 metres), Xiangyang County (XY, Hubei Province,longitude: E 112.21, latitude: N 32.08; the average altitude is 13 metres), Fuzhou City (FZ, Fujian Province, longitude: E 119.28, latitude: N 26.08; the average altitude is 600~1000 metres) .

Access & import/export

Describe the efforts you have made to access habitats and to collect and import/export your samples in a responsible manner and in compliance with local, national and international laws, noting any permits that were obtained (give the name of the issuing authority, the date of issue, and any identifying information).

Disturbance

Describe any disturbance caused by the study and how it was minimized.

Reporting for specific materials, systems and methods

We require information from authors about some types of materials, experimental systems and methods used in many studies. Here, indicate whether each material, system or method listed is relevant to your study. If you are not sure if a list item applies to your research, read the appropriate section before selecting a response.

Materials & experimental systems

n/a	Involved in the study	
☐	☒	Antibodies
☒	☐	Eukaryotic cell lines
☒	☐	Palaeontology and archaeology
☒	☐	Animals and other organisms
☒	☐	Clinical data
☒	☐	Dual use research of concern

Methods

n/a	Involved in the study	
☒	☐	ChIP-seq
☒	☐	Flow cytometry
☒	☐	MRI-based neuroimaging

Antibodies

Antibodies used

PO8-VHH nanoantibody that was produced by our laboratory; the sheep-HRP-IgG anti-rabbit (Lot number: BA1054; Boster Biological Technology co.ltd);rabbit polyclonal antibody that was produced by our laboratory.

Validation

The sheep-HRP-IgG anti-rabbit is HRP Conjugated AffiniPure Goat Anti-rabbit IgG (H+L).
